# Association of miR-223-3p and miR-223-5p gene expression with levels of IgE, vitamin D and Mg+2 in pediatric asthma patients

**DOI:** 10.5937/jomb0-54841

**Published:** 2025-11-05

**Authors:** Duaa F. Al-Mashhadani, Basima Q. Alsaadi

**Affiliations:** 1 Ministry of Health, Baghdad, Iraq; 2 University of Baghdad, Institute of Genetic Engineering and Biotechnology for Post-graduate Studies, Baghdad, Iraq

**Keywords:** pediatric asthma, miR-223-3p, miR-223-5p, IgE, vitamin D, Mg+2, dečja astma, miR-223-3p, miR-223-5p, IgE, vitamin D, Mg+2

## Abstract

**Background:**

Asthma, a global non-communicable disease, significantly impacts public health. Severe cases cause high morbidity and mortality rates, with childhood asthma rates in Iraq reaching 20%. The study investigated the expression levels of miR-223-3p and miR-223-5p genes linked to pediatric asthma and the impact of IgE, Vitamin D, and Mg+2 on asthma severity.

**Methods:**

A study of 140 children aged 1-10 years with allergic asthma was conducted in Baghdad from November 2023 to February 2024. The patients were divided into three groups: those under 1, those aged 1-5, and those over 5. Serum IgE, Vit D3, and Mg+2 levels were determined using the Immunoglobulin E Test Kit.

**Results:**

The results indicated a significant increase in the level of IgE in patients (353.812 ± 2 5 .679 ng/mL) compared to the control group (25.320± 2.581 ng/mL) (pvalue &lt; 0.01, the serum level of Vit-D revealed a significant decrease in the patient group (16 .9 0 7 ± 0 .5 1 2 ng/mL) while in the control group, levels reached (32.746± 1.629 ng/mL), Mg+2 serum level decreased in patient groups (2 .1 6 8 ± 0 .0 3 0 ng/dL) compared to control group (2 .316± 0.028 ng/dL) at p-value &lt; 0.01. also, this study shows that upregulation of gene expression in miR-223-3p and miR-223-5p genes are considered risk factors for allergic asthma.

**Conclusions:**

The study found that asthma patients had high IgE levels, low vitamin D3 and magnesium levels, and high gene expression in miR-223-3p and miR-223-5p genes, risk factors for allergic asthma.

## Abstract (sr)

**Uvod:**

Astma je globalna nezarazna bolest koja značajno utiče na javno zdravlje. Teski oblici astme uzrokuju visoku stopu morbiditeta i mortaliteta, pri čemu stopa dečje astme u Iraku dostiže 20%. Ova studija istraživala je nivoe ekspresije gena miR-223-3p i miR-223-5p povezanih sa dečjom astmom, kao i uticaj IgE, vitamina D i Mg+2 na težinu astme.

**Metode:**

Studija je obuhvatila 140 dece uzrasta od 1 do 10 godina sa alergijskom astmom, a sprovedena je u Bagdadu od novembra 2023. do februara 2024. godine. Pacijenti su podeljeni u tri grupe: mlađi od 1 godine, uzrasta od 1 do 5 godina i stariji od 5 godina. Nivoi IgE, vitamina D3 i Mg+2 u serumu određeni su korišćenjem kompleta za testiranje imunoglobulina E.

**Rezultati:**

Rezultati su pokazali značajno povećanje nivoa IgE kod pacijenata (353,812± 25,679 ng/mL) u poređenju sa kontrolnom grupom (25,320± 2,581 ng/mL) (p&lt; 0,01). Nivo vitamina D u serumu je bio značajno snižen kod pacijenata (16,907± 0,512 ng/mL) u poređenju sa kontrolnom grupom (32,746± 1,629 ng/mL). Nivo Mg+2 u serumu bio je niži kod pacijenata (2,168± 0,030 ng/dL) u odnosu na kontrolnu grupu (2,316± 0,028 ng/dL) uz p &lt; 0,01. Takođe, studija je pokazala da je povećana ekspresija gena miR-223-3p i miR-223-5p povezana sa povećanim rizikom od razvoja alergijske astme.

**Zaključak:**

Studija je utvrdila da pacijenti sa astmom imaju visoke nivoe IgE, niske nivoe vitamina D3 i magnezijuma, kao i povećanu ekspresiju gena miR-223-3p i miR-223-5p, što predstavlja faktore rizika za razvoj alergijske astme.

## Introduction

Allergic asthma is a prevalent chronic inflammatory airway disorder characterised by various clinical manifestations and intricate genetic and causative variables [1] [2].

Allergic asthma is represented by airway inflammation, hyper-responsiveness, increased immunoglobulin (Ig) E levels, airway remodelling, and clinical manifestations including wheezing, dyspnea, chest tightness, cough, and airflow obstruction [3]. In 2020, the World Health Organization (WHO) estimated that approximately 339 million individuals had asthma, with the majority of fatalities occurring among older adults. According to the Global Initiative for Asthma (GINA), asthma affects 1% to 18% of populations across various countries, and its prevalence has risen globally [3] [4].

Immunoglobulin E (IgE) is pivotal in the pathogenesis of asthma and other allergic conditions, including urticaria and allergic rhinitis. One idea regarding the rising incidence of asthma pertains to vitamin D (VitD). Specific individuals contend that many variables linked to westernisation have contributed to diminished (VitD) levels, leading to increased asthma prevalence.7.8 Conversely, some contend that Vitamin D exerts a more detrimental influence on allergy aetiology [5] [6]. Magnesium (Mg^+2^) is a cofactor regulating several enzymatic and cellular activities. Another advantageous impact in asthma is reduced acetylcholine release from cholinergic neurons and diminished histamine release from mast cells. Enhanced bronchial smooth muscle contractility leading to bronchial hyper-reactivity is a defining pathophysiological event of asthma [7]. Magnesium is a crucial factor in the contraction and relaxation condition of the bronchial smooth muscle. Magnesium positively influences bronchial asthma through various mechanisms, including direct bronchodilation and functioning as a natural calcium antagonist [8]. Research indicates that Mg^+2^ treats severe asthma [9] [10]. Research on serum Mg^+2^ concentrations in asthma patients indicates that hypomagnesemia is prevalent in individuals with asthma [11]. MicroRNAs (miRNAs) are valuable diagnostic, prognostic, and therapeutic biomarkers for asthma. miRNAs are endogenous noncoding RNAs, approximately 19 to 25 nucleotides in length, involved in post-transcriptional gene regulation. Numerous miRNAs have been identified as contributors to lung formation, immunological responses, and many pulmonary illnesses, including lung cancer, asthma, COPD, and pulmonary fibrosis [12]. Prior research has demonstrated that various miRNAs, such as miR-223-3p and miR-223-5p, exhibit upregulation or downregulation in individuals with asthma and correlate with increased adverse events, significant symptom burden, or accelerated decline in lung function [13]. Various cell types generate miRNAs, which are released into serum/plasma, saliva, bronchoalveolar lavage fluid (BALF), and other bodily fluids. Moreover, miRNAs are consistently found in circulating blood and other bodily fluids, where they perform biological functions via fluid circulation and serve as non-invasive biomarkers for diagnosing many disorders, including asthma. Recently, increased focus has been directed towards multifunctional miR-223 because of its crucial involvement in the immune system [14]. The correlation between miR-223-3p expression in the peripheral blood leukocytes of asthmatic patients and inflammatory cytokines is not yet elucidated [15]. There are many studies in Iraq about the relationship between genetics and asthma such as [4] [16] [17] [18] [19] [20] [21] [22]. The aim of the current study is to investigate the relationship between gene expression and also the levels of Vitamin D3, IgE, and Mg^+2^ with severity of asthma.

## Materials and methods

### Subject

The current study was conducted on 140 subjects, 60 control and 80 patients (52 females and 88 males) with 1-10 years of allergic asthma. The patients were separated into three groups: The first group consisted of those under 1 year old, the Second group included those aged 1-5 years, and the third group included those beyond 5 years old, who were admitted to the Central Children's Hospital, Al-Kadhimiya Children's Hospital, Al-Alawiya Children's Hospital, and Al-Zahraa Center for Asthma and Allergy in Baghdad over the period from November 2023 to February 2024.

### Blood sample collection

Five mL of venous blood was collected using a sterile syringe. Divide this blood into 3 mL, place it in a gel tube, and leave it for 20 minutes to clot at room temperature (25-30°C). The tubes were then centrifuged at 3000 RPM over 15 minutes to separate the serum, and the serum was then stored in Eppendorf tubes at -20°C until used for immunohistochemical assay. The remaining 2 mL of blood was placed in an EDTA tube. The remaining 2 mL of blood was placed in an EDTA tube. Then, 250 μL of blood sample was added to 750 μL in Trizol Eppendorf and frozen at -20°C for molecular analysis.

### Determination of vitamin D3

A solitary assessment of VitD, quantified as 25-hydroxy cholecalciferol, 25(OH)D, was conducted in all participants utilising a chemiluminescent technique (Liaison 25-OH Vitamin D Total; Diasorin, Saluggia, Italy). Values were treated as continuous variables, whereas vitamin D was classified in descriptive analyses as desirable (or acceptable) at levels of at least 30 to 40 ng/mL (75 to 100 nmol/L), insufficient between 20 and 30 ng/mL (50 and 75 nmol/L), and deficient when below 20 ng/mL.

### Determination levels of [Mg] in serum

Magnesium concentrations in biological samples were measured using the DRI-CHEM NX500i, an automated dry chemistry analyser developed by Fujifilm. The device is known for its high accuracy and ease of use, making it ideal for rapid and efficient analysis of clinical samples. The DRI-CHEM NX500i operates on the principle of dry chemistry, using reactive strips embedded with specific chemical reagents that interact with Mg^+2^ in the sample. The serum sample is introduced into the device, and the analysis is automatically performed. After processing the data, the device provides accurate results for Mg^+2^ concentration in the appropriate units.

### Determination of serum IgE levels

### Immunoglobulin E test kit (Rate Scattering Turbidimetric Method)

The IgE assay is used by modified dispersion turbidimetry to determine serum IgE levels to assess immune function and adjunctive diagnosis of immune diseases. IgE in the sample is bound to a specific antibody, which causes light to scatter and is proportional to IgE levels. A specialised device measures the intensity of the scattered light, and IgE concentrations are estimated by comparing the turbidity of the sample to a standard concentration. Results are recorded and repeated as needed, with control checks performed at the beginning of each batch to ensure accuracy and comparison of results to reference values.

### Molecular study

### Genomic RNA extraction

RNA was isolated from the blood of patients with asthma and a control group of apparently healthy individuals using the TransZol Up Plus RNA Kit (blood). The RNA content and purity were subsequently assessed using a Nanodrop spectrophotometer from the business Transgen.

### cDNA synthesis for mRNA

The EasyScript® One-Step gDNA Removal and complementary DNA (cDNA) Synthesis SuperMix kit was used to reverse-transcribe total RNA into cDNA. Following the manufacturer's guidelines, the reaction volume was set at 20 μL, utilising 20 μL of total RNA for the conversion process. as shown in Table 1.

**Table 1:** Strand cDNA synthesis reaction component.

Component	volume reaction
mRNA/miRNA	4 μL
Anchored Oligo(dT)18<br>Primer (0.5 μg/μL)	1 μL
Random Primer<br>(0.1 μg/μL)	1 μL
GSP	1 μL/10 μmol
2xES Reaction Mix	10 μL
*EasyScript* ® RT/RI<br>Enzyme Mix	1 μL
gDNA Remover	1 μL
RNase-free Water	1 μL
**Total volume**	**20 μL**

### Gene expression of MiR-223-3P and MiR-223-5P by quantitative real-time PCR (qRT-PCR)

Total RNA was reverse transcribed into complementary DNA (cDNA) with the EasyScript One-Step gDNA Removal and cDNA Synthesis SuperMix Kit from TransGen Biotech Co., China, in a reaction volume of 20 μL adhering to the manufacturer's instructions. The quantitative real-time PCR (qRT-PCR) was performed utilising the QIAGEN Rotor-Gene Q real-time PCR system (Germany). Each qRT-PCR reaction involved 2 μL of cDNA, 1 μL for both the forward and reverse primers (with a concentration of 10 μmol/L) as listed in Table 2, and 10 μL of the PerfectStartTM. The green qPCR SuperMix kit from TransGen Biotech Co., China. The thermal profile consisted of an initial step at 94°C for 5 minutes (one cycle), followed by 40 cycles involving denaturation at 94°C for 5 minutes, annealing at 58°C for MiR-223-3P MiR-223-5P and miR-U6 for 15 seconds, and extension at 72°C for 20 seconds. The final dissociation stage spanned from 55 to 95°C, with each degree lasting 5 seconds. The specificity of the amplified product was confirmed through melting curve analyses. The relative expression of the MiR-223-3P and MiR-223-5P genes in the study group samples was evaluated by normalising their expression levels to the reference gene miR-U6 using the Ct1 method [20]. The data were presented as the fold change in MiR-223-3P and MiR-223-5P gene expression compared to the healthy controls within the study groups. This allowed for normalising the expression levels against the reference gene miR-U6. The median fold expression of MiR-223-3P and MiR-223-5P in the study groups was then utilised to assess whether there were statistically significant differences in MiR-223-3P and MiR-223-5P gene mRNA expression levels Significant differences.

**Table 2:** Primer sequences were utilised in this study's assays. *Ta: annealing temperature

**Primer**	**Sequence (5'→3' direction)**	**primer size**<br>**(bp)**	**T_a_** <br>**(°C)**	**Design in the current study**
***miRNA***				
MiR-223-3P	TGTCAGTTT GTCAAATACCCCA	22	58	
MiR-223-5P	CGTGTATTTGACAAGCIGAGTT	22		
miRNA-universe R.P.	GCGAGCACAGAATTAATACGAC	22		
Universe R.transcription p	CAGGTCCAGTTTTTTTTTTTTTTTVN	26		
MiR -U6	AGAGAAGATTAGCATGGCCCCT	22	58	

### MiR-223-3P and MiR-223-5P gene expression calculation

The fold changes in the quantitative expression of mature RNAs were assessed using the relative cycle threshold (2-ΔΔCt) method, initially introduced by Livak and Schmittgen in 2001. It is the ratio of relative gene expression between the control group and the experimental group. The double delta Ct (threshold cycle) analysis was used to assess the expression of *MiR-223-3P* and *MiR-223-5P* genes, the housekeeping reference genes. The calculations were as follows: The real-time cycler software calculated the threshold cycle (CT) for each sample. The samples were duplicated, and the average results were computed. The Ct values for the target genes MiR-223-3P and MiR-223-5P which were being evaluated in both patients and controls, were reported.

The ΔCt, or difference in CT values, also called the »normalised raw data,« was determined by subtracting the specified normalisation factor from the Ct value of each target gene and the housekeeping gene.

ΔCt (control)=CT (gene)-CT(HKG)

ΔCt (patient)=CT (gene)-CT(HKG) ΔΔCt=ΔCt (patient)-ΔCt (control).

### Ethical approval

The study used the ethical principles outlined in the Declaration of Helsinki. The study was performed following the acquisition of both verbal and written consent from the patients before collecting the samples. This case-control study was approved by the Scientific Committee of the Institute of Genetic Engineering and Biotechnology for past graduate study at the University of Baghdad, and the study was approved by the Ministry of Health and Environment of Iraq (4778 on 25-12-2023).

### Statistical analysis

The results of the present study were analysed related to the objectives and presented according to the general description of the sample. Microsoft Excel 2010 and SPSS (version 25) software were used for statistics analysis. Microsoft package (Excel and Word). The data are expressed as mean ± SD, and differences were considered significant when p-values were *P*<0.05.

## Results

### Serum IgE, vitamin D and Mg^+2^ levels in studied groups

**Figure 1:**
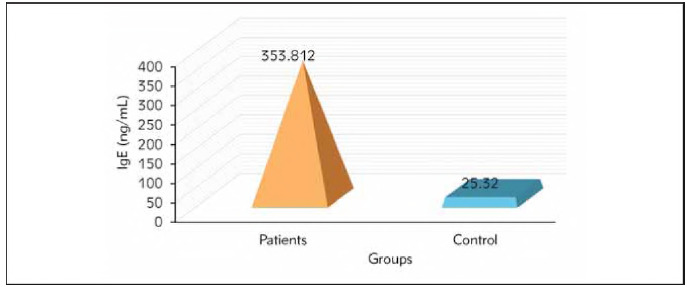
Comparison between the IgE(ng/mL) levels between patients and control groups.

**Figure 2:**
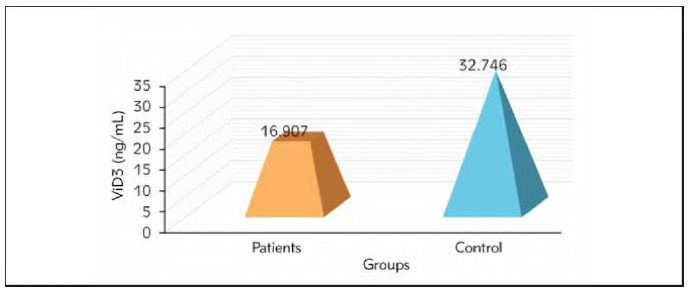
Comparison between the Vit.D3 between patients and control groups.

The current study showed the levels of IgE in the blood serum of both patients and control groups. The results indicated a significant increase in the level of IgE in patients (353.812±25.679 ng/mL) compared to the control group (25.320±2.581 ng/mL) (p-value <0.01) as shown in Figure 1. The serum level of Vit-D revealed a significant decrease in the patient group (16.907±0.512 ng/mL), while in the control group, levels reached (32.746±1.629 ng/mL), as shown in Figure 2.

### The miR-223-3p and miR-223-5p gene expression

The results demonstrated that using miR-U6 for normalisation in qRT-PCR is a highly reliable approach, particularly in clinical studies. Furthermore, the 2-ct value and the ratio of 2-ct for various study groups compared to the control group were employed to assess changes in miR-U6 expression across different study groups, as presented in Table 3. The 2^-ΔΔCt^ value for asthma patients was 304.7537; for control, it was 258.078. The calculated fold expression ratios for the gene were 1.180 and 1.00, respectively. These variations in gene fold expression among the study groups underscore the utility of the miR-U6 gene as a reliable control. The expression of the miR-223-3p gene showed highly significant differences (p<0.001) in the asthma patient group compared to the control group. Similarly, Table 4 shows the 2^-ΔΔCt^ value for asthma patients was 18.600, and for control, it was 8.141. The calculated fold expression ratios for the gene were 2.29 and 1.00, respectively. The expression of the *miR-223-5p* gene showed highly significant differences (p<0.001) in the asthma patient group compared to the control group.

**Table 3:** Comparison between patients and healthy control groups regarding miR-223-3p fold expression levels.

Groups	Means Ct<br>ofmiR223-3p	Means Ct<br>ofU6	ΔCt (Means Ct<br>of miR223-3p)	2-ΔCt	experimental group/<br>Control group	Fold of gene<br>expression
**Patients**	16.4761	24.7276	-8.2515	304.7537	304.7537/258.078	1.180
**Control**	16.2341	24.2458	-8.01166	258.078	258.078/258.078	1.00

**Table 4:** Comparison between patients and healthy control groups regarding miR-223-5p fold expression levels.

Groups	Means Ct<br>ofmiR223-5p	Means Ct<br>ofU6	ΔCt (Means Ct<br>of miR223-5p)	2-ΔCt	experimental group/<br>Control group	Fold of gene<br>expression
**Patients**	20.5103	24.7276	-4.21725	18.600	18.600/8.141	2.29
**Control**	21.2371	24.2625	-3.0252	8.141	8.141/8.141	1.00

### Quantification of miR-223-3p expression by real-time PCR

**Figure 3:**
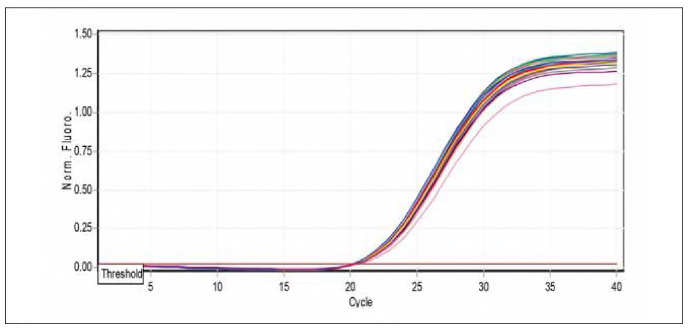
Amplification curve chart miR-223-5p gene.

**Figure 4:**
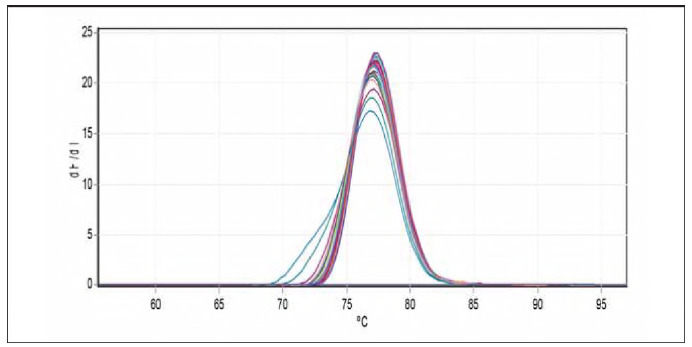
Melting curve chart miR-223-3p gene.

The qPCR samples of the studied groups determined miRNA-223-3p dissociation curves. Table 3 shows the results of miRNA-223-3p levels in children and patients infected with allergic asthma, and healthy controls are demonstrated in Table 3. The folding of miRNA-223-3p (1.180) revealed higher levels in patients with allergic asthma and [1] in apparently healthy controls, as shown in Figure 3 and Figure 4.

### Quantification of miR-223-5p expression by real-time PCR

**Figure 5:**
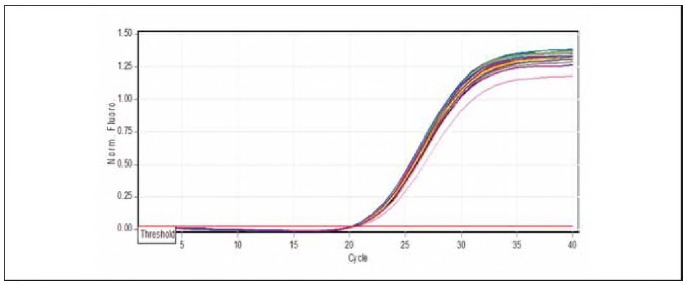
Amplification curve chart miR-223-5p gene.

**Figure 6:**
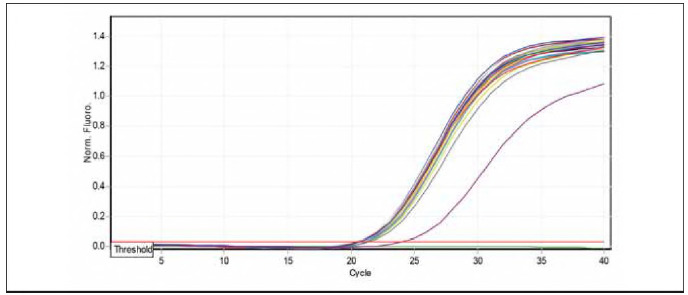
Melting curve chart miR-223-5p gene.

miRNA-223-5p dissociation curves by q PCR samples of studied groups. The results of miRNA-223-5p levels in children patients infected with allergic asthma and healthy controls are demonstrated in Table 4. The folding of miRNA-223-5p (2.29) revealed higher levels in patients with allergic asthma and [1] in apparently healthy controls, as shown in Figure 5 and Figure 6).

In Table 5, the Pearson correlation between miR-223-3p and miR-223-5p showed a strong positive correlation of 0.659 with a statistical significance of less than 0.001. This correlation suggests a possible co-regulatory relationship or involvement in a specific biological pathway, which explores their role in childhood asthma and its associated biological mechanisms.

**Table 5:** Pearson correlations for miR-223-3p and miR-223-5p. **Correlation is significant at the 0.001 level (2-tailed).*

		miR223-3p	miR223-5p
miR223-3p	Pearson<br>Correlation	1	.659**
	Sig. (2-tailed)		<.001
miR223-5p	Pearson<br>Correlation		1
	Sig. (2-tailed)		

## Discussion

Immunoglobulin E (IgE) plays a crucial role in allergic diseases, particularly asthma, due to its heightened sensitivity to allergens; hence, measuring IgE levels aids in diagnosing asthma and monitoring patient status. The present study showed elevated total serum IgE of asthmatic compared to control at (P<0.001). The result agrees with previous studies [23]. Another study by [24] indicates that the mean serum IgE level was 554 IU/mL in asthmatic patients, while that of the control was 69 IU/mL. Further, the present results agree with a previous international study, Stromgaard et al. [25] identified a positive correlation between total blood IgE levels and asthma in patients . Elevations in IgE levels may be attributed to viral factors (the predominant cause of asthma symptom aggravation), specific allergens, or may merely indicate a broad elevation of IgE synthesis. Consequently, IgE levels can be utilised to distinguish between asthmatic and non-asthmatic individuals [26]. The current study showed a decrease in vitamin D in apathetic patients compared to control. These findings are consistent with the results obtained from other studies, both adults and children [27].

The active form of vitamin D can inhibit inflammation by enhancing the secretion of anti-inflammatory cytokines and chemokines. So, vitamin D's endocrine, autocrine, paracrine, and immune-modulating activity emerged in the body [28]. Another study demonstrated that incorporating VitD3 supplementation into inhaled corticosteroid therapy for asthma did not significantly impact the duration until severe asthma developed or asthma-related morbidity [29]. Furthermore, VitD supplementation did not markedly decrease the incidence of initial treatment failure. Additionally, no observable effects on asthma control, pulmonary function, asthma symptoms, quality of life, or airway inflammation were noted [30].

In this study, the serum Mg^+2^ level of asthmatic children was significantly lower compared to the control group. In agreement with [31] Lytvynets LI. [32] that there was no significant difference in serum levels between asthmatics and controls; nevertheless, they discovered that asthmatics had lower levels of intracellular magnesium in their erythrocytes. His discovery may offer medicinal advantages. Magnesium therapy for asthmatic patients may enhance clinical outcomes, as indicated by [33]. Low Mg^+2^ levels may, according to a study by Jebur and Saud [6], be associated with worsening asthma symptoms, with patients having lower levels than healthy controls. These findings are consistent with the current study, as evidence suggests that Mg^+2^ deficiency may play a role in worsening asthma symptoms and is a potential factor influencing disease progression [8]. It is possible that the clinical condition of asthmatic patients could be improved by using Mg^+2^ supplements. Anti-inflammatory drugs, also known as glucocorticoids, and bronchodilator agents, also known as beta-2 agonists, are among the medications utilised to treat asthma. Patients who use these medications for an extended period may experience a decrease in magnesium levels due to urine excretion and intracellular shift [34].

MiR -U6 is one of the most commonly employed housekeeping genes for assessing gene expression data [35]. In a study conducted by Robert and colleagues [36], the expression of 1,718 genes across 72 different types of normal human tissues was investigated using quantitative real-time polymerase chain reaction (qRT-PCR), with miR *-U6* serving as a reference gene.

This study showed increased Mir-223-3p expression in severe asthma cases in children. This is consistent with the previous study's findings [15], which found that the expression levels miR-223-3p in peripheral blood leukocytes were significantly higher in asthma patients than in healthy children. Some results have been reached by another study (2016), which indicated an increase in the expression of the Mir-223-3p, miR-629-3p, and miR142-3 genes, indicating the possibility of its contribution to the exact inflammatory mechanism related to Increased expression of mir223-3p is considered as a biomarker in the diagnosis of allergic asthma in children. A study conducted by Xu et al. [15] on the development of asthma through several genetic and molecular analyses clarified the role of mir-223-3p in developing and exacerbating the disease in childhood. Numerous studies have shown significant shifts in miR-223 expression during the development of inflammatory states. This predisposition points to plausible significant functions of this miRNA in maintaining a balanced inflammatory state [37]. A study similar to the current study showed that the most critical biological study of miR-223-3p pertains to cilium construction and organisation in bronchial epithelial cells, facilitating mucociliary function and mucus clearance, hence reducing infections and inflammation [38]. miR-223-3p is involved in the inflammatory response, and its expression level significantly changes in children with allergic asthma. Therefore, it is vital to explore the correlation between miR-223-3p and allergic asthma [39]. Recent investigations into asthma have shown that exosomes play a part in the development of asthma. The results showed that the exosome derived from neutrophil swarm 1 contains miR-223-3p, which helps delay the inflammatory response in asthma [40]. In addition, airway-derived miR-223-3p suppresses the expression of TNF- -stimulated gene 6 in peripheral white blood cells, promoting asthma development [41]. These results demonstrate that miR-223-3p plays a dual role in regulating the inflammatory response associated with asthma.

This study showed a significant increase in the expression levels of miR-223-5p, consistent with previous studies. As explained by earlier data [42], they conducted experiments on mouse models of asthma and revealed a similar increase in the expression of miR-223-5p in the affected models. MiR-223-5p is involved in various autoimmune disorders in children. Micro RNA223-5p may aim at an immunosuppressive effect to control inflammation or remain up-regulated to favour disease progression and susceptibility to infection MiR-223 [43] by driving the switch between effective regulatory cells of B and T cells and dendritic cells, may contribute to immune interaction and control immunomodulation [44]. The importance of miR-223-5p in asthma pathogenesis and disease exacerbation has received significant attention in asthma [45]. Recent research has shown that the miR-223 family of microRNAs, including Mir223-5p, which is highly selectively expressed in granulocytes, is critical in controlling neutrophil function [46]. The major function of miR-223-5p is to repress gene expression at the post-transcriptional level. In macrophages, statistical histogram analysis and analysis have identified possible miR-223-5p target genes involved in the inflammatory response, such as Ccl3, Tnf, Cxcl2, and Lcn2. A condition known as a microvascular complications-independent asthma predictor enhances miR-223-5p expression via the p38 [47]. This shows that miR-223-5p expression is more closely linked with asthma than miR-223-3p. The conclusion reached was that miR-223 was involved in lung protection from chronic inflammation and played a crucial role in those mechanisms that developed within an inflammatory process, thereby in diseases such as asthma [38].

## Conclusion

Current results indicate that serum the levels of IgE in the blood serum showed a significant in the patients compared to the control; the serum level of Vit-D revealed a considerable decrease in the patient group than in the control group, and magnesium levels are lower in asthmatics, and depending on the patients' magnesium condition, these variations were substantial. In addition, the *miR-223-3p* and *miR-223-5p* may be regarded as risk factors for the onset of asthma in the Iraqi population. To validate these results, larger sample sizes and additional research are required.

## Dodatak

### Acknowledgements

The authors want to thank and appreciate the staff of the Institute of Genetic Engineering and Biotechnology for Post Graduate Studies, University of Baghdad, for their help and support.

### Conflict of interest statement

All the authors declare that they have no conflict of interest in this work.
